# Electrochemical Stability of Electrodeposited PEDOT:PSS Neural Stimulation Electrodes

**DOI:** 10.3390/mi17101198

**Published:** 2026-10-17

**Authors:** Amirhossein Azami, Yupeng Wu, Christopher K. Nguyen, Justin R. Abbott, Mark E. Orazem, Kevin J. Otto, Stuart F. Cogan, Brian N. Kim

**Affiliations:** 1 Department of Electrical Engineering and Computer Science, University of Texas at Dallas, Richardson, TX 75080, USA; amirhossein.azami@utdallas.edu; 2 Department of Material Science and Engineering, University of Texas at Dallas, Richardson, TX 75080, USA; wyp@uoregon.edu; 3 Department of Bioengineering, University of Texas at Dallas, Richardson, TX 75080, USA; christopher.nguyen@utah.edu (C.K.N.); jabbott@blackrockneuro.com (J.R.A.); stuart.cogan@utdallas.edu (S.F.C.); 4 Department of Chemical Engineering, University of Florida, Gainesville, FL 32611, USA; morazem@che.ufl.edu; 5 Weldon School of Biomedical Engineering, Purdue University, West Lafayette, IN 47907, USA; kotto@purdue.edu

**Keywords:** neural electrodes, neural stimulation, electrode coatings, poly(3,4-ethylenedioxythiophene):polystyrenesulfonate (PEDOT:PSS), electrochemical stability, long-term stimulation

## Abstract

Microelectrode arrays are used for neural stimulation and recording; however, their performance is limited by the high impedance and low charge-injection capacity of conventional metallic interfaces. To overcome these challenges, a variety of coatings, including metal oxides, carbon nanotubes, and conductive polymers, are applied to improve the electrochemical performance of microelectrode arrays. Among these materials, poly(3,4-ethylenedioxythiophene):polystyrene sulfonate (PEDOT:PSS) has gained significant attention due to its low impedance, high charge-injection capacity, and compatibility with microfabrication processes. Despite these advantages, the long-term electrochemical stability of PEDOT:PSS at high levels of stimulation charge injection remains insufficiently understood. In this study, we investigated the stability of PEDOT:PSS films electrodeposited on gold microelectrodes for prolonged electrical stimulation. Voltage transient (VT) analysis revealed that PEDOT:PSS degradation occurs when the maximum cathodic excursion (E_mc_) exceeds –0.6 V versus Ag|AgCl. The degradation is accompanied by the appearance of asymmetric access voltages and a voltage inversion in the anodal phase of the VT. Electrochemical impedance spectroscopy (EIS) revealed a sharp increase in PEDOT:PSS impedance at negative DC bias potentials, beginning at −0.5 V and increasing as the potential became more negative. These results identified –0.6 V as an electrochemical limit for PEDOT:PSS stability on gold and implicate the reduction and de-doping of PEDOT:PSS, and sequalae, in the observed PEDOT:PSS degradation. Nonetheless, PEDOT:PSS electrodes had stable charge-injection capacities of 3.6 mC/cm^2^ (200 µs pulse width) over the course of 200 million pulses.

## 1. Introduction

High spatial precision electrical stimulation for neuromodulation is advancing rapidly, with microfabrication techniques leading to the creation of microelectrode arrays that enable high-resolution neurostimulation [1,2,3]. However, further miniaturization to achieve even higher spatial resolution is limited by the inherently high impedance of metallic microelectrodes [4,5]. The characteristically high impedance of metallic microelectrodes arises because charge transfer is confined to the available electrode surface through the electrical double layer and pseudocapacitive processes. Miniaturization to microelectrode dimensions reduces the available electrochemical surface area, with a consequent high impedance and low charge-injection capacity. PEDOT:PSS, functioning as a mixed ionic–electronic conductor, permits ionic penetration throughout the hydrated, porous polymer matrix, enabling the charge to be exchanged volumetrically across the full depth of the film, rather than being restricted to the electrode surface. This volumetric charge-exchange mechanism markedly increases both the effective electrochemical surface area and the interfacial capacitance, ultimately reducing the electrode impedance by several orders of magnitude relative to the bare metal of the equivalent geometric area. The high impedance increases the likelihood of irreversible water oxidation and reduction during neurostimulation, which are potentially damaging to both the microelectrode and surrounding tissue. Electrode coatings can be used to reduce the impedance and increase the charge-injection capacity of microelectrodes. Such coatings include metal oxides like iridium oxide and ruthenium oxide [6,7,8], titanium nitride [9], intrinsically conductive polymers such as poly(3,4-ethylenedioxythiophene) (PEDOT) [10,11,12,13] and carbon nanotube or graphene oxide composites with PEDOT [14,15,16]. Besides low impedance, high charge-injection capacity, and the potential for biofunctionalization [17,18], conductive polymers have attracted attention as microelectrode coatings due to their ease of incorporation into electrode arrays by direct electropolymerization onto metallization at electrode sites [19]. Beyond conventional metallic and thin-film microelectrodes, PEDOT-based interfacial coatings are increasingly being applied to emerging soft and stretchable bioelectronic platforms. Notable examples include liquid-metal-based electrodes, where such coatings have demonstrated the ability to simultaneously reduce interfacial impedance and enhance both electrochemical performance and mechanical stability [20,21].

Many useful reviews of conductive polymer chemistry and PEDOT-based electrode coatings are available, including [22,23,24,25,26]. The electronic conductivity and electrostatic stability of PEDOT are obtained by introducing a counterion dopant into its structure [24,27]. Doped PEDOT is a mixed conductor, exhibiting both electronic conductivity, specifically hole conductivity, and ionic conductivity, stemming from the conjugated structure of the polymer chain [28]. The PSS^-^ anion is probably the most extensively employed counterion used with PEDOT in neural applications, although many other counterions have been explored and shown to influence the structure and potentially improve the stability of doped PEDOT [29,30,31]. Previous studies have shown that PEDOT:PSS provides a high signal-to-noise ratio and excellent neural recording quality [32,33], and high charge-injection capacity with stability suitable for chronic neural stimulation [34,35,36]. For example, Cui et al. [11] observed a 10–20-fold increase in charge-storage capacity (CSC), a two-order magnitude decrease in impedance, and a maximum charge injection capacity (CIC) of 2.3 mC/cm^2^ for PEDOT:PSS-coated electrodes compared to uncoated Au and Pt electrodes. Venkatraman et al. [13] demonstrated PEDOT:PSS stability over 24 h of pulsing (8.5 million pulses, CIC = 3 mC/cm^2^) by comparing changes in CSC and impedance before and after pulsing. Wilks et al. [36] examined the stability of PEDOT:PSS under short-term current pulsing (CIC = 5 mC/cm^2^, 720,000 pulses). Their study assessed impedance and CSC before and after stimulation, revealing a small reduction in CSC and stable impedance. More recently, Oldroyd et al. demonstrated the stability of PEDOT:PSS over 1.4 billion stimulation pulses at a charge-injection level of 0.3 mC/cm^2^ [37].

Although previous studies have demonstrated PEDOT:PSS stability during current pulsing, the electrochemical limits of stability have not been explored in detail. In the present work, we evaluated the stability of PEDOT:PSS electropolymerized on gold microelectrodes, focusing on the relation between the negative potential excursion and stability during cathodal-first, biphasic current pulsing. Our results demonstrate that PEDOT:PSS subjected to 200 million pulses maintained electrochemical functionality when pulsed cathodally first at charge densities ≤ 3.6–4 mC/cm^2^, corresponding to a maximum negative potential excursion (E_mc_) of −0.6 V Ag|AgCl. However, pulsing at higher charge densities, resulting in an E_mc_ more negative than −0.6 V, induced degradation. The degradation was associated with the appearance of an inversion in the anodal phase of the voltage transient, which is attributed to increasing PEDOT:PSS impedance in the leading cathodal phase at potentials more negative than −0.6 V. These findings underscore the importance of avoiding charge-injection levels that polarize PEDOT:PSS coatings, at least those polymerized directly on gold microelectrodes, to potentials more negative than −0.6 V vs. Ag|AgCl.

## 2. Materials and Methods

This section describes the substrate preparation, microfabrication, and the PEDOT:PSS electrodeposition. The electrochemical characterization included cyclic voltammetry, voltage transients, and impedance spectroscopy. The pulsing strategy, morphological and structural analysis, and statistical methods are presented.

### 2.1. Gold Substrate Preparation and Microfabrication

**Figure 1:**
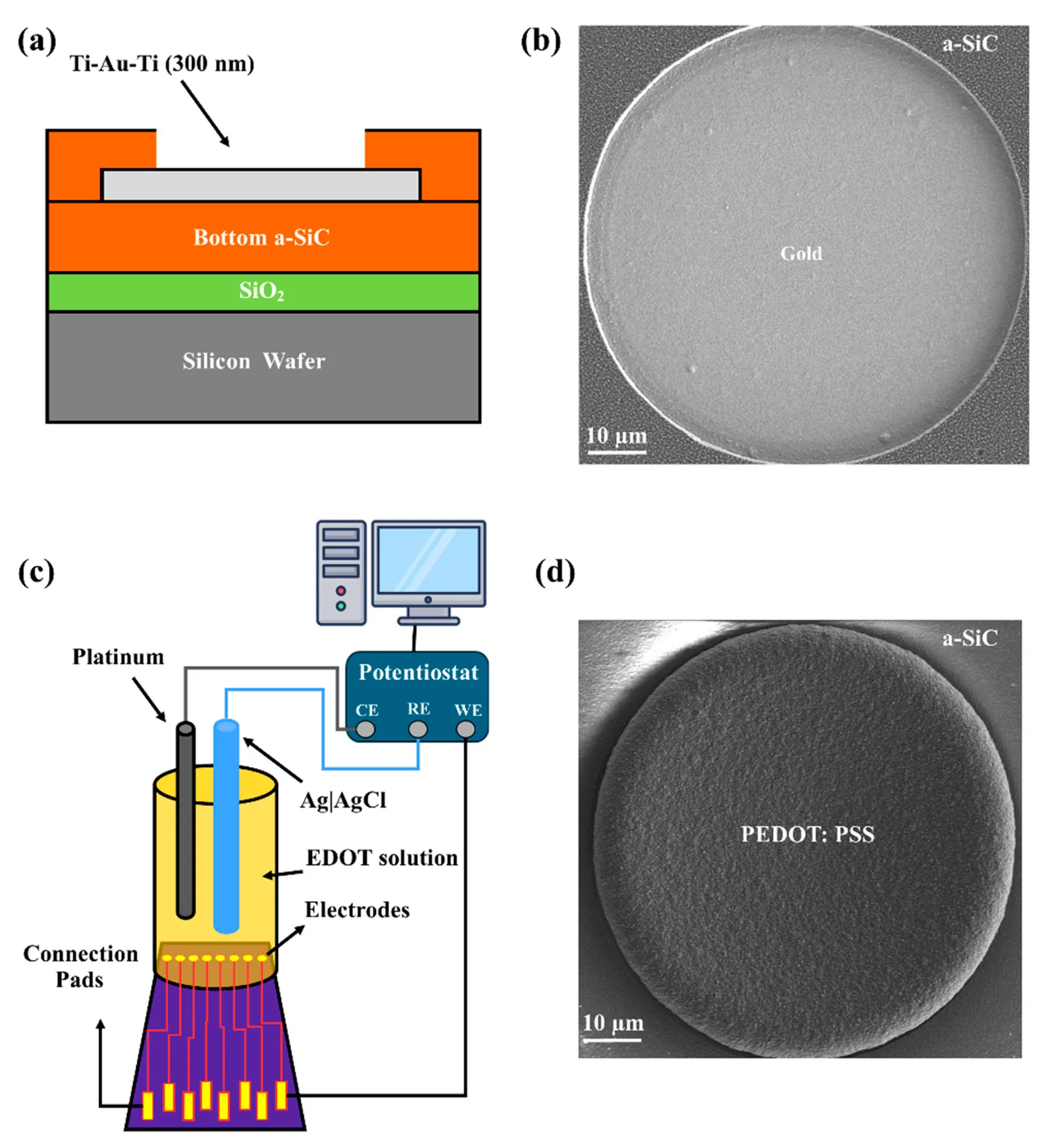
Device design and electrodeposition setup: (**a**) cross-sectional schema of an electrode site; (**b**) SEM of 2000 µm^2^ gold site prior to PEDOT:PSS coating; (**c**) electrodeposition cell comprising a Pt counter, Au working, and Ag|AgCl reference electrodes in the EDOT/PSS deposition solution; (**d**) SEM of 2000 µm^2^ gold site after PEDOT:PSS deposition.

PEDOT:PSS was electropolymerized onto gold microelectrodes with a geometric surface area (GSA) of 2000 µm^2^. Arrays of the gold microelectrodes were fabricated on 100 mm silicon wafers. Each wafer was divided into four quarters, with each quarter containing 36 electrodes: 32 functional sites and four blank channels without exposed gold. A cross-sectional view of the multilayer structure of the electrode sites, designed to interface with external electrochemical test equipment, is shown in Figure 1a. The fabrication process begins with the thermal oxidation of the silicon wafer to form a 1 µm thick SiO_2_ insulating layer, followed by the deposition of a 2 µm thick amorphous silicon carbide (a-SiC) layer using a PlasmaTherm Vision 310 plasma-enhanced chemical vapor deposition (PECVD) system (PlasmaTherm, Saint Petersburg, FL, USA). Subsequently, a conductive stack of three metal layers (Ti/Au/Ti: thickness 50/200/50 nm) was deposited onto the a-SiC using a Temescal E-beam deposition system (Temescal Systems, Livermore, CA, USA). This metal stack was patterned into metal traces using photolithography followed by wet etching (BOE, 7:1) and potassium iodine (Transense, Danvers, MA, USA). A second 2 µm thick a-SiC layer was then deposited over the metal traces and the first a-SiC layer. Photolithography and dry etching (Sirius-T2 RIE Etcher, Trion Technology, Clearwater, FL, USA) were performed to expose the bond pads and electrode sites and to remove the top Ti adhesion layer, exposing the gold for PEDOT deposition. A representative SEM image, shown in Figure 1b, reveals a uniform gold electrode surface.

### 2.2. PEDOT:PSS Electrodeposition

Before electropolymerization, the gold electrode surfaces were cleaned by rinsing with isopropyl alcohol and acetone, followed by rinsing with deionized water. Subsequently, prior to deposition, the electrodes were subjected to a 30 min oxygen-plasma treatment using a reactive ion etcher (March Asher, PX-250, Nordson March, Westlake, OH, USA) with oxygen plasma (27 Pa, 50 watts). The plasma treatment served the dual purpose of cleaning and lightly roughening the gold surface. The electrodeposition solution comprised 50 mM EDOT, 50 mM polyoxyethylene-10-lauryl ether, and 100 mM NaPSS (all MilliporeSigma, Burlington, MA, USA). For deposition, a three-electrode electrochemical cell was assembled, comprising the gold working electrode, a platinum wire counter electrode, and a Ag|AgCl reference electrode (Figure 1c). Potentiodynamic electropolymerization by cyclic voltammetry, using a Gamry Interface 1000E potentiostat (Gamry Instruments, Warminster, PA, USA), was used for PEDOT:PSS deposition, similar to methods reported by others [11,38] and reviewed in [19]. Each gold electrode underwent 50 CV cycles over a potential range of 0 to 0.9 V vs. Ag|AgCl. Fifty CV cycles were sufficient to deposit uniform PEDOT:PSS films with high cathodal CSC_c_ (Figure 1d).

### 2.3. Electrochemical Characterization

PEDOT:PSS coated electrodes were characterized by EIS and CV in phosphate-buffered saline (PBS) at 37 °C using the three-electrode cell described previously. The PBS had a conductivity of 21–23 mS/cm and pH of 7.1–7.3. EIS was performed over a frequency range of 0.05 Hz to 100 kHz using a 10 mV rms sinusoidal signal, typically around open circuit potential (E_oc_). EIS spectra were also collected as a function of DC potential bias from −0.9 to 0.6 V to assess impedance behavior as a function of potential. For each biased measurement, the designated bias was applied to the film for 10 min, and the EIS was recorded immediately thereafter at the bias potential. CVs were acquired at 50 mV/s between limits of −0.9 V and 0.6 V vs. Ag|AgCl, corresponding to the reported water reduction and oxidation potentials on PEDOT:PSS [1]. The CSC_c_ of the PEDOT:PSS was determined over the −0.9 to 0.6 V potential range from the time integral of the cathodal current during a CV cycle.

### 2.4. Pulsed and Passive Stability

To assess the pulsing stability of PEDOT:PSS, VTs were recorded for up to 200 million pulses in argon-sparged PBS at 37 °C using a two-electrode configuration, with a stainless steel (SS) electrode serving as the counter electrode and pseudo-reference electrode. Stainless steel was selected as the pseudo-reference electrode primarily because its open-circuit potential (E_oc_) remained relatively close to 0 V versus Ag|AgCl (when measured, its E_oc_ remained within a +50 to –50 mV range from 0 V). Cathodal-first, symmetric biphasic current pulses with a 200 µs phase duration, 20 µs interphase delay, and 500 Hz pulse frequency were employed (Plexstim, Plexon Inc., Dallas, TX, USA). E_mc_ was determined as a function of current amplitude and defined as the electrode potential. E_mc_ was measured 12 µs after the end of the leading cathodal current phase. An E_mc_ range of −0.5 to −0.9 V was selected for VT tests based on previous reports of increased film impedance within this range [1]. For current pulsing, the current amplitude was incrementally increased in 50 µA steps until the E_mc_ reached the desired value for pulsing. Electrode stability was assessed for up to 200 million pulses at the desired E_mc_ by periodically monitoring changes in 1 kHz impedance, CSC_c_, and VT response. The charge per phase for each pulsing study required occasional minor adjustments during pulsing to maintain a constant E_mc_.

The passive stability of PEDOT:PSS in 37 °C argon-sparged PBS was assessed using five electrodes electropolymerized as above for pulsed electrodes. The 50 mV/s CVs, 50,000 mV/s CVs, and EIS were measured initially and periodically over a 5-day duration, matching the time used in the long-term pulsing experiments to reach 200 million pulses.

### 2.5. Morphological and Structural Analysis

The morphology of the PEDOT:PSS was imaged by scanning electron microscopy (SEM) using a Zeiss SIGMA 500 VP microscope (Carl Zeiss Microscopy, White Plains, NY, USA).

### 2.6. Statistical Methods

GraphPad Prism version 10.4.1 (GraphPad Software, Boston, MA, USA) was used for statistical analysis and creating graphical representations of the results. Unless otherwise noted, data with repetitions are reported as mean ± standard deviation.

## 3. Results

**Figure 2:**
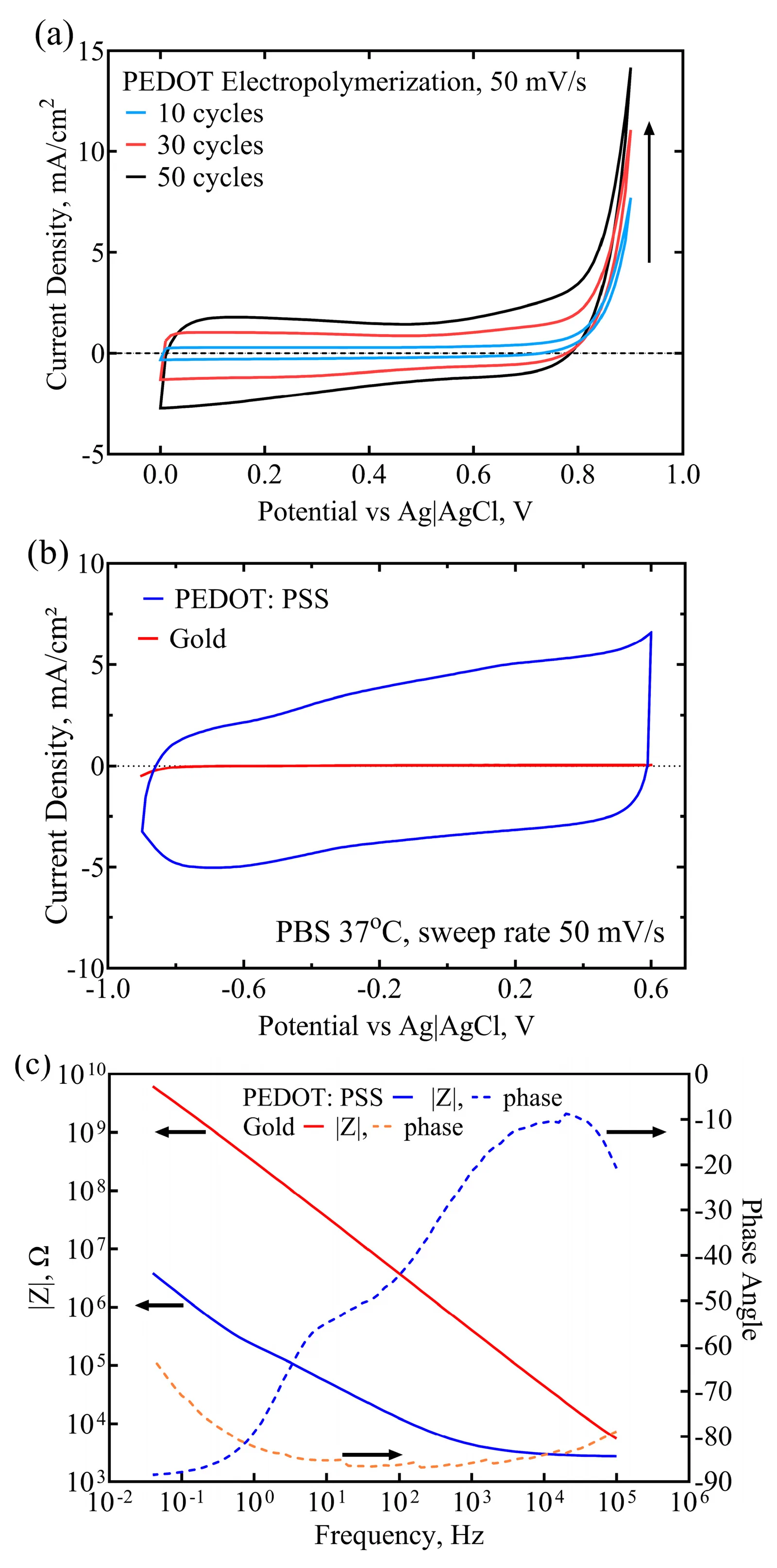
Electropolymerization cyclic voltammograms and electrochemical characterization of PEDOT:PSS. (**a**) Electropolymerization CVs for 10, 30, and 50 cycles, showing increasing charge capacity with number of deposition cycles, where the arrow indicates the increase in enclosed charge with successive cycles and the horizontal dashed line marks zero current density; (**b**) 50 mV/s CV of 2000 µm^2^ PEDOT:PSS compared to an uncoated gold electrode, with the horizontal dashed line again marking zero current density; (**c**) EIS impedance comparison of a PEDOT:PSS coated and uncoated gold electrode site, in which solid lines with square and circular markers show the impedance magnitude (|Z|, left axis) and dashed lines with triangular markers show the phase angle (right axis), the arrows indicating the axis to which each data set corresponds. GSA = 2000 µm^2^ for all electrodes.

As shown in Figure 1d, SEM images revealed complete PEDOT:PSS coverage of the gold sites. The surface morphology of the PEDOT:PSS exhibited a granular structure with a slight elevation along the circumference of the electrode, which is attributed to a nonuniform current distribution during electropolymerization. The as-deposited PEDOT:PSS extended approximately 3 µm beyond the via diameter. This circumferential annulus does not directly contact the exposed gold in the via and its contribution to the electrochemical properties of the electrode is uncertain. For this reason, electrode GSAs were based on the 50 µm via diameter which is precisely defined during thin-film fabrication. A PEDOT:PSS thickness of 1–2 µm at the via circumference was estimated by imaging the edges of delaminated, but intact, electrodes that had not been electrochemically tested. However, the thickness was variable, appearing thinner at the center of the electrodes, and the near-plan view angle of the images precluded precise thickness measurement. The granular morphology increases the electrochemical surface area of PEDOT-based conductive polymers, contributing to reduced impedance and increased CIC, as discussed by Zheng et al. [39]. The CV behavior during electropolymerization is shown in Figure 2a. The arrow indicates the progressive expansion of the area enclosed by the deposition CVs as the number of cycles increases, corresponding to a gradual thickening of the PEDOT:PSS film. For all PEDOT:PSS electrodes, the cathodic and anodic charge capacities at the 50th deposition cycle ranged from 24 to 29 mC/cm^2^ and 30 to 39 mC/cm^2^, respectively. The higher anodal charge capacity must, in part, be due to EDOT oxidation during electropolymerization, although water oxidation may also contribute to the anodal charge. A representative CV of PEDOT:PSS in 37 °C PBS at a 50 mV/s sweep rate is shown in Figure 2b, with a CV of the uncoated gold site prior to deposition for comparison. The CSC_c_ of the PEDOT:PSS (50 electropolymerization cycles) was 93 mC/cm^2^ compared with 0.6 mC/cm^2^ for the uncoated gold. Overall, the CSC_c_ of uncoated gold sites increased from 0.6 ± 0.35 mC/cm^2^ to 94 ± 6 mC/cm^2^ (*n* = 4). The deposition limit of 50 CV cycles was necessary to avoid delamination, as PEDOT films deposited with more than 50 cycles, while exhibiting high CSC_c_ (>100 mC/cm^2^), often showed poor adhesion to the gold substrate. Although increasing film thickness enhanced both the charge-storage and charge-injection capacities, frequent delamination at the gold–PEDOT interface precluded studies with thicker films. We suspect the larger volumetric changes encountered during reduction or oxidation of the thicker PEDOT:PSS is the cause of delamination, but this has not been confirmed. Similarly, the PEDOT:PSS impedance magnitude exhibited a marked reduction post-deposition, as shown in Figure 2c, in which 1 kHz impedance decreased from 300 kΩ to 4.6 kΩ. Overall, PEDOT:PSS deposition resulted in an impedance reduction of 99.8% at 1 Hz (102 ± 11.4 MΩ to 0.23 ± 0.02 MΩ), 98.4% at 1 kHz (301 ± 28 kΩ to 4.7 ± 0.3 kΩ), and 81% at 30 kHz (15 ± 0.9 kΩ to 2.9 ± 0.2 kΩ) (*n* = 4). The electrodes selected for electrochemical characterization were sourced from a minimum of three independently fabricated wafer pieces. The reported means and standard deviations (*n* = 4) therefore reflect sample-to-sample variability arising across independent fabrication and deposition runs, rather than localized variation within a single array. Changes in phase angle, shown in Figure 2c, reflect a shift from the predominantly pseudocapacitive behavior of the gold to the more faradaic behavior of the PEDOT:PSS.

**Figure 3:**
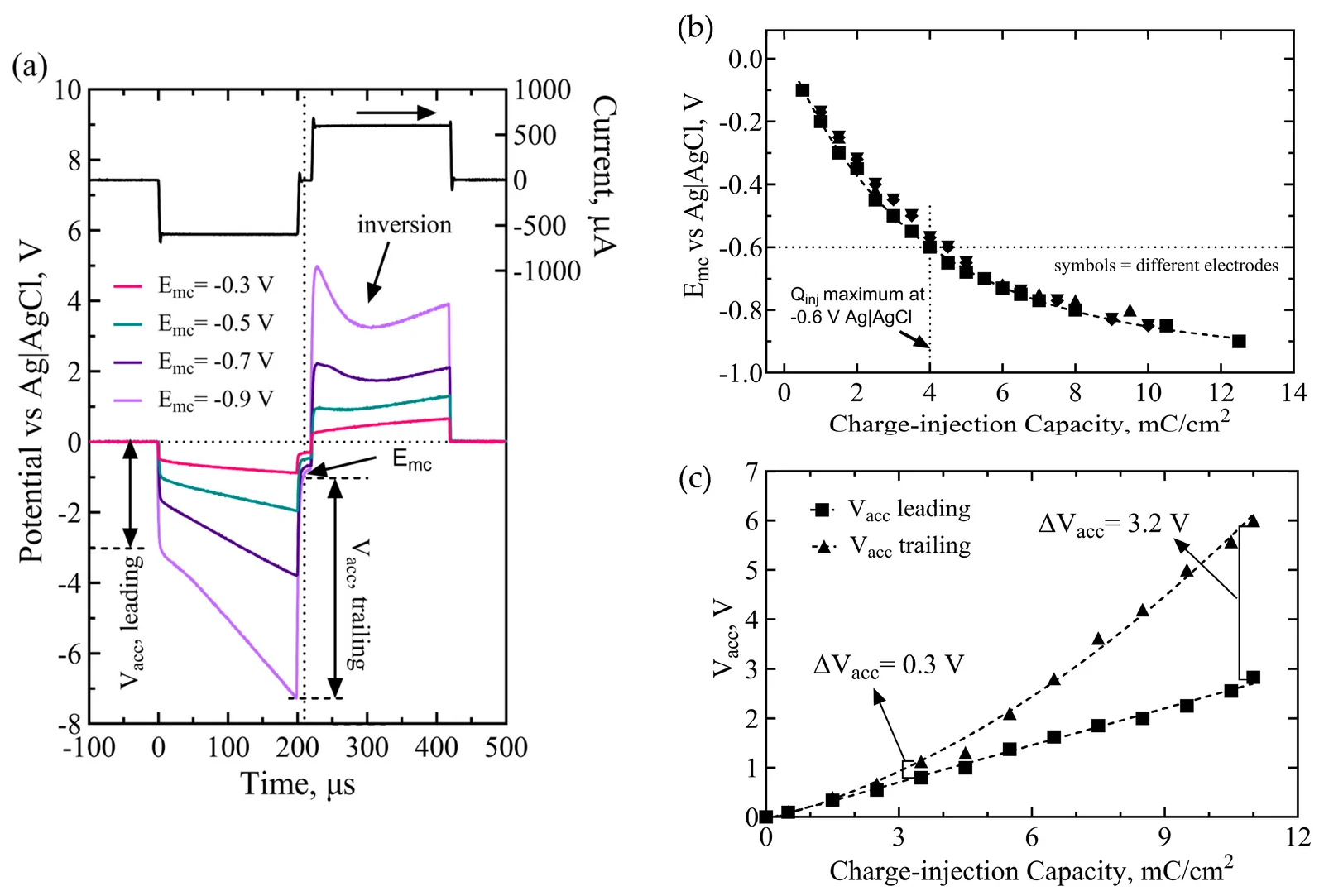
Current pulsing of PEDOT:PSS: (**a**) voltage transient response of PEDOT:PSS pulsed to increasingly negative E_mc_ with biphasic, charge-balanced, cathodal-first current pulses (phase duration 200 µs, interphase delay 20 µs, frequency of 500 Hz). The arrow shows an inversion in the anodal phase of the VT for E_mc_ values of −0.5 V and more negative; (**b**) E_mc_ as a function of Q_inj_; (**c**) progression of ∆V_acc_ between the leading and trailing V_acc_ as a function of Q_inj_. The asymmetry becomes apparent at Q_inj_ > 3.6 mC/cm^2^. Dashed lines are drawn as an aid to the eye.

The VT response of the PEDOT:PSS was characterized by pulsing to negative E_mc_ potentials between −0.1 V and −0.9 V. VTs of a PEDOT:PSS electrode, with a pre-pulsing CSC_c_ of 81 mC/cm^2^, for selected E_mc_ values are shown in Figure 3a. The relationship between Q_inj_ and E_mc_ is shown in Figure 3b and suggests a maximum CIC of ~12 mC/cm^2^ at E_mc_ = −0.9 V for the electrode shown. The average maximum Q_inj_ for each E_mc_, averaged over the four electrodes tested, is listed in Table 1. A maximum Q_inj_ of 12±1 mC/cm^2^ was obtained at −0.9 V (*n* = 4), similar to that previously reported for PEDOT:PSS pulsed to E_mc_ = −0.9 V [4].

**Figure 4:**
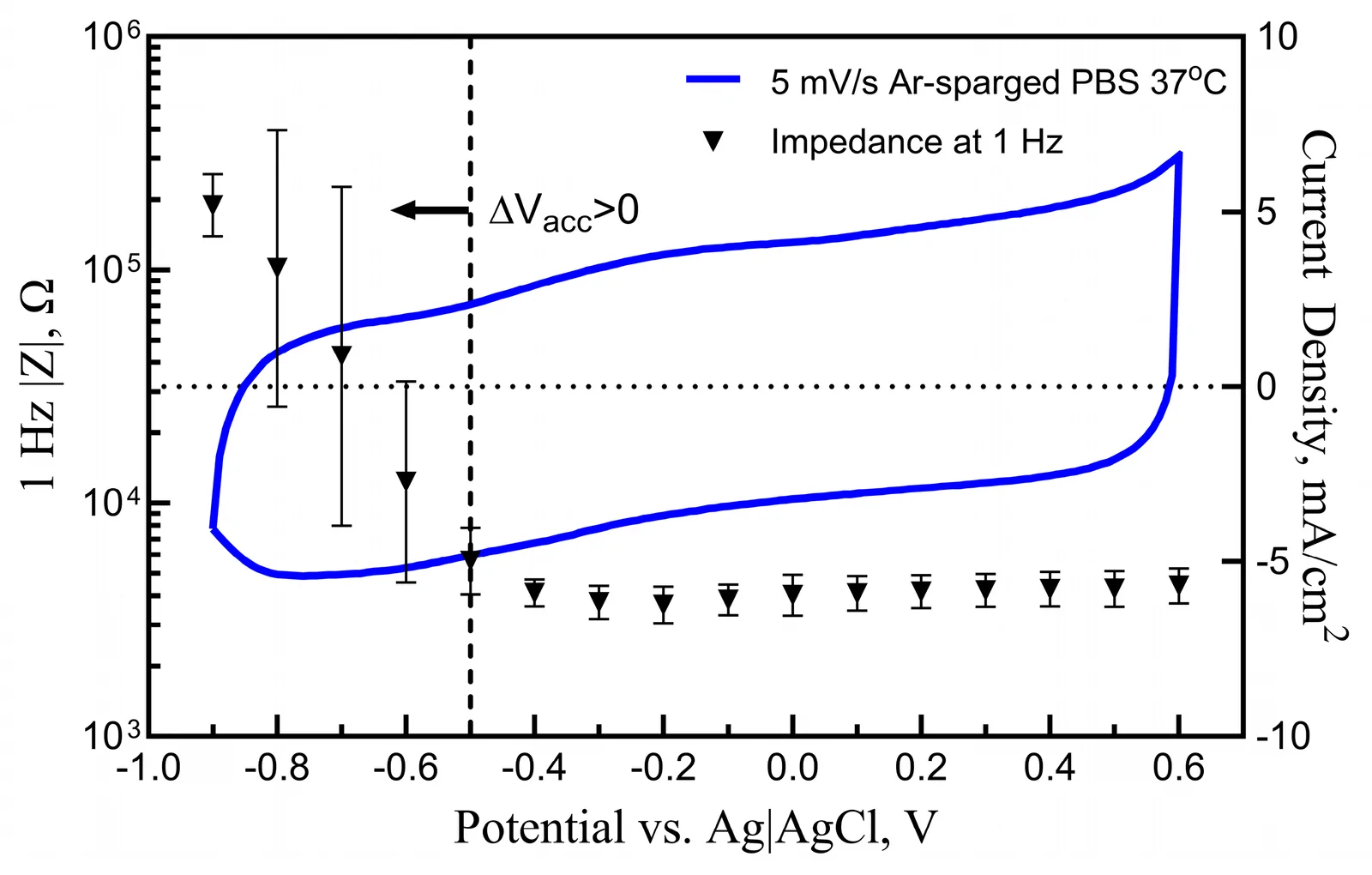
Steady-state PEDOT:PSS impedance at 1 Hz plotted for different potential bias levels, superimposed over a 5 mV/s CV. The vertical dashed line at −0.5 V Ag|AgCl shows the potential at which impedance increases and ΔV_acc_ > 0 V.

A notable phenomenon observed in the VTs was an asymmetry in the leading and trailing access voltages (V_acc_) (defined in Figure 3a). The magnitude of the trailing V_acc_ becomes larger than that of the leading V_acc_ for Q_inj_ between 3.6–4 mC/cm^2^, corresponding to an E_mc_ of approximately −0.6 V, as shown in Figure 3c. Although an increase in PEDOT:PSS impedance is apparent in near-steady-state EIS measurements (Figure 4), the asymmetry in V_acc_ only becomes apparent at an E_mc_ of −0.6 V, presumably due to the more limited reduction of the PEDOT:PSS in the −0.5 V transient. At higher Q_inj_ levels, up to 11 mC/cm^2^ at E_mc_= −0.9 V, the difference in leading and trailing access voltages reaches 3.2 V, reflecting a substantial increase in the resistive component of the impedance during the cathodal current phase. This potential-dependent increase in PEDOT:PSS impedance is evident in Figure 4, which shows an overlay of 1 Hz impedance magnitude on a 5 mV/s CV. The 1 Hz impedance in Figure 4 was selected because at this frequency the electrochemical response, as shown by the EIS spectrum in Figure 2c, includes faradaic contributions which capture the reduction reactions leading to decreased PEDOT:PSS conductivity during stimulation pulsing. The 1 kHz impedance is reported elsewhere to facilitate comparison with impedance values reported in the broader literature [40,41]. At potentials negative of −0.5 V, the impedance increases from a baseline of ~2 × 10^5^ Ω at −0.5 V to >1 × 10^7^ Ω at −0.9 V. Although the film impedance begins to increase at potentials more negative than approximately −0.5 V, coatings pulsed to a maximum cathodic potential (E_mc_) of −0.6 V remained fully functional and electrochemically stable over the course of 200 million pulses. The observed rise in impedance therefore reflects the onset of progressive film reduction rather than the onset of irreversible failure, the latter of which was observed exclusively at E_mc_ values of −0.7 V or more negative. Additionally, an inversion in the voltage transient response was observed during the anodal phase of the biphasic pulse, as shown by the arrow in Figure 3a. This inversion first becomes apparent at charge densities for which E_mc_ is driven to −0.5 V Ag|AgCl and becomes increasingly apparent at more negative potentials. The inversion is characterized by a low driving voltage over the initial portion of the anodal phase. The effect becomes more pronounced at higher charge densities and, correspondingly, more negative E_mc_. It should be noted that the potential-dependent impedance changes reported in Figure 4 will be larger than those observed at equivalent E_mc_ values during pulsing because only a portion of the PEDOT:PSS film is reduced during the 200 µs cathodal pulse.

**Figure 5:**
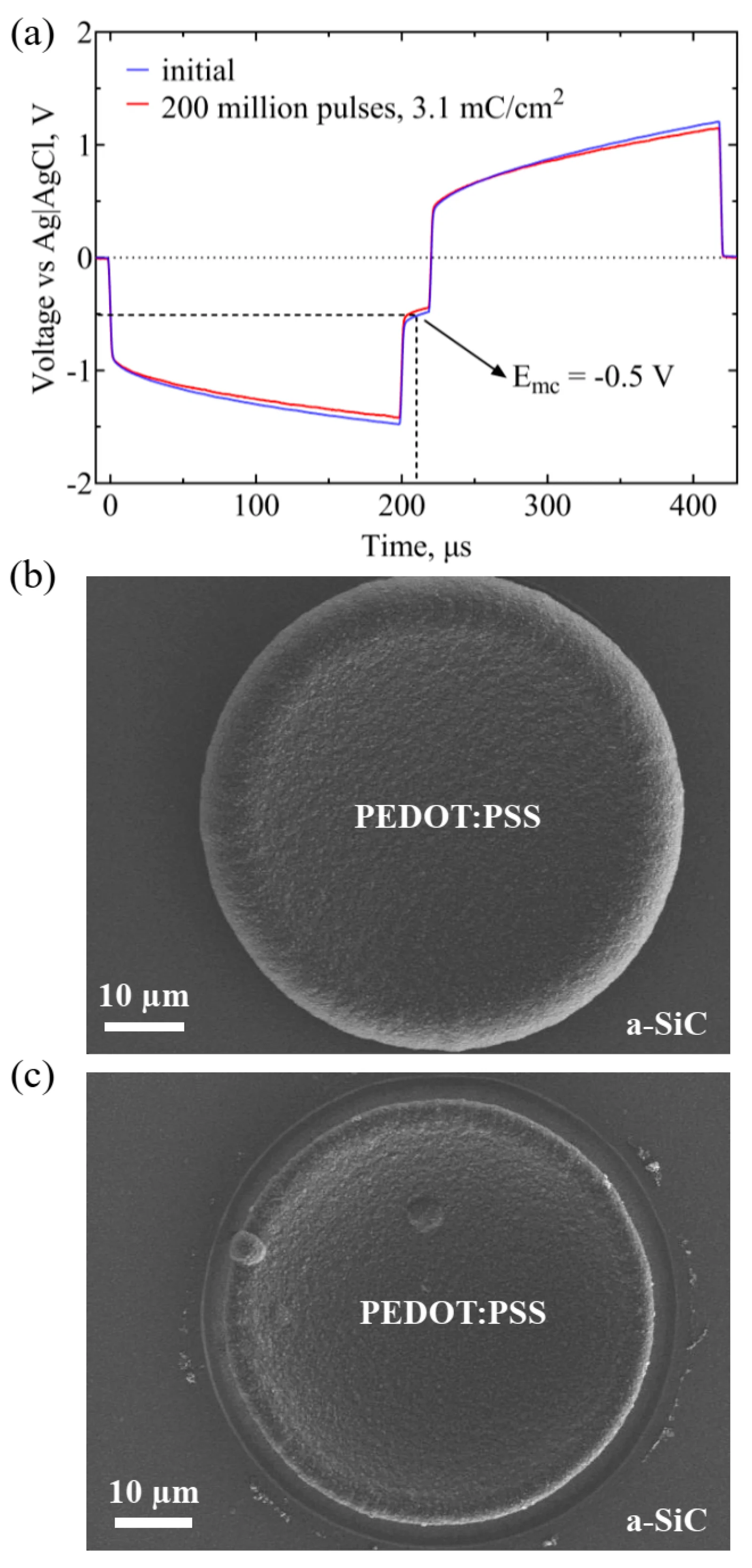
(**a**) VT response of PEDOT:PSS pulsed to an E_mc_ = −0.5 V, before and after 200 million pulses, where the dotted horizontal line marks 0 V and the dashed lines indicate E_mc_ measured at the end of the cathodal current phase; (**b**) SEM of an unpulsed PEDOT:PSS electrode; (**c**) SEM of PEDOT:PSS after 200 million pulses to E_mc_ = −0.5 V. The physical structure of the film remained intact, with post-stimulation electrochemical characterization showing only modest changes in the film’s electrochemical characteristics.

To assess stability, PEDOT:PSS electrodes were subjected to current pulsing at E_mc_ values from −0.5 to −0.9 V, and the CSC_c_, 1 kHz impedance, and VTs monitored for up to 200 million biphasic current pulses. PEDOT:PSS electrodes consistently tolerated 200 million pulses without failure when pulsed to E_mc_ = −0.5 V or E_mc_ = −0.6 V. Changes in the VT response, shown in Figure 5a, were minimal. SEM imaging of an unpulsed electrode site, Figure 5b, and a site subjected to 200 million pulses at E_mc_ = −0.5 V, Figure 5c, showed that PEDOT:PSS coatings remained substantially intact when pulsed to E_mc_ = −0.5 V, corresponding in Figure 5a to a Q_inj_ of 3.1 mC/cm^2^. Similarly, PEDOT:PSS films pulsed to E_mc_ = −0.6 V, corresponding to a Q_inj_ of 3.6 mC/cm^2^ (for a film with CSC_c_ of 73 mC/cm^2^) were stable for 200 million pulses.

**Figure 6:**
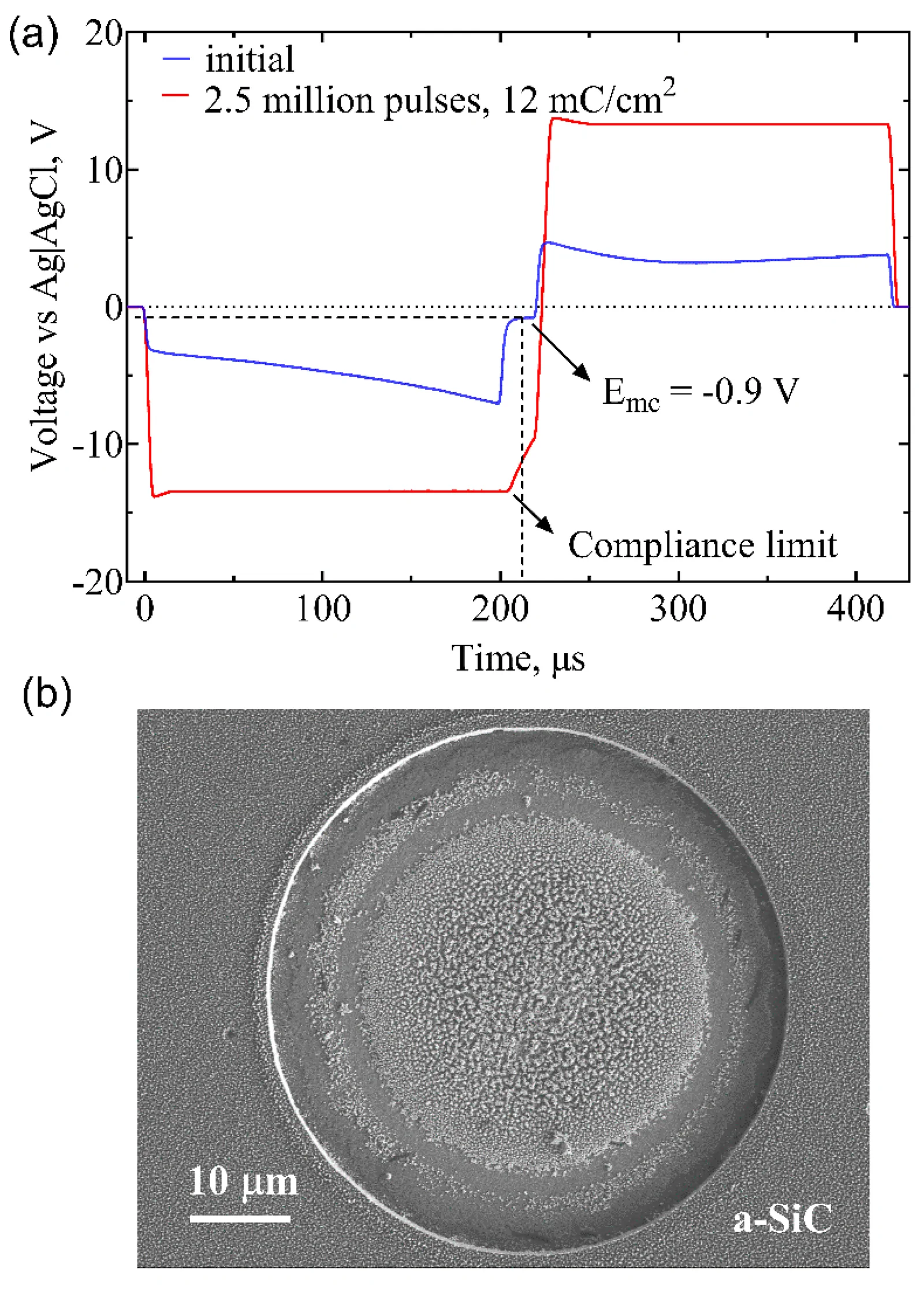
(**a**) VT of PEDOT:PSS pulsed to E_mc_ = −0.9 V before and after 2.5 million pulses, where the dotted horizontal line marks 0 V and the dashed lines indicate E_mc_ measured at the end of the cathodal current phase. The voltage compliance limit of the stimulator is reached when attempting to pulse to −0.9 V after delamination of the PEDOT:PSS; (**b**) SEM of PEDOT:PSS after 2.5 million pulses to E_mc_ = −0.9 V, showing delamination and loss of the PEDOT:PSS coating.

However, long-term pulsing to an E_mc_ ≤ −0.7 V led to the failure of three of the four electrodes tested, with only one electrode reaching 200 million pulses. This observation highlights the intrinsic variability in the number of pulses to failure and that E_mc_ thresholds for failure will depend on the maximum pulse number employed in stability testing. PEDOT:PSS electrodes pulsed at E_mc_ = −0.7 V and −0.8 V survived for an average of 174 ± 36 and 48 ± 39 million pulses (*n* = 4), respectively. Failure of the PEDOT:PSS was characterized by a large decrease in CSC_c_, from 90 ± 15 mC/cm^2^ to 12 ± 19 mC/cm^2^ for electrodes pulsed to −0.7 V and from 97 ± 36 mC/cm^2^ to 0.54 mC/cm^2^ for pulsing to −0.8 V (Table 1). Similarly, VTs recorded before and after pulsing to E_mc_ = –0.9 V, shown in Figure 6a, revealed degradation after only 2.5 million pulses. Failure was characterized by a complete loss of PEDOT:PSS accompanied by a large reduction in CSC_c_ and an increase in impedance. The electrode surface after degradation for an electrode pulsed to E_mc_ = −0.9 V (12 ± 1 mC/cm^2^) shows only a thin residue of material remaining on the electrode surface (Figure 6b). The nature of the residue was not determined.

PEDOT:PSS remained stable when passively soaked in PBS (37 °C, nitrogen sparge) over five days, equivalent to the time required for 200 million pulses at 500 Hz. The slow-sweep-rate CV profiles remained consistent, indicating minimal degradation. Quantitatively, the charge-storage capacity (CSC_c_) changed by less than 1% (from 78.8 ± 2.8 mC/cm^2^ to 77.4 ± 1.6 mC/cm^2^), and the impedance at 1 kHz decreased by approximately 17% (from 4.1 ± 0.13 kΩ to 3.4 ± 0.1 kΩ). These findings indicate that passive exposure to PBS at 37 °C does not contribute to significant degradation of PEDOT:PSS during pulsed stability testing.

**Figure 7:**
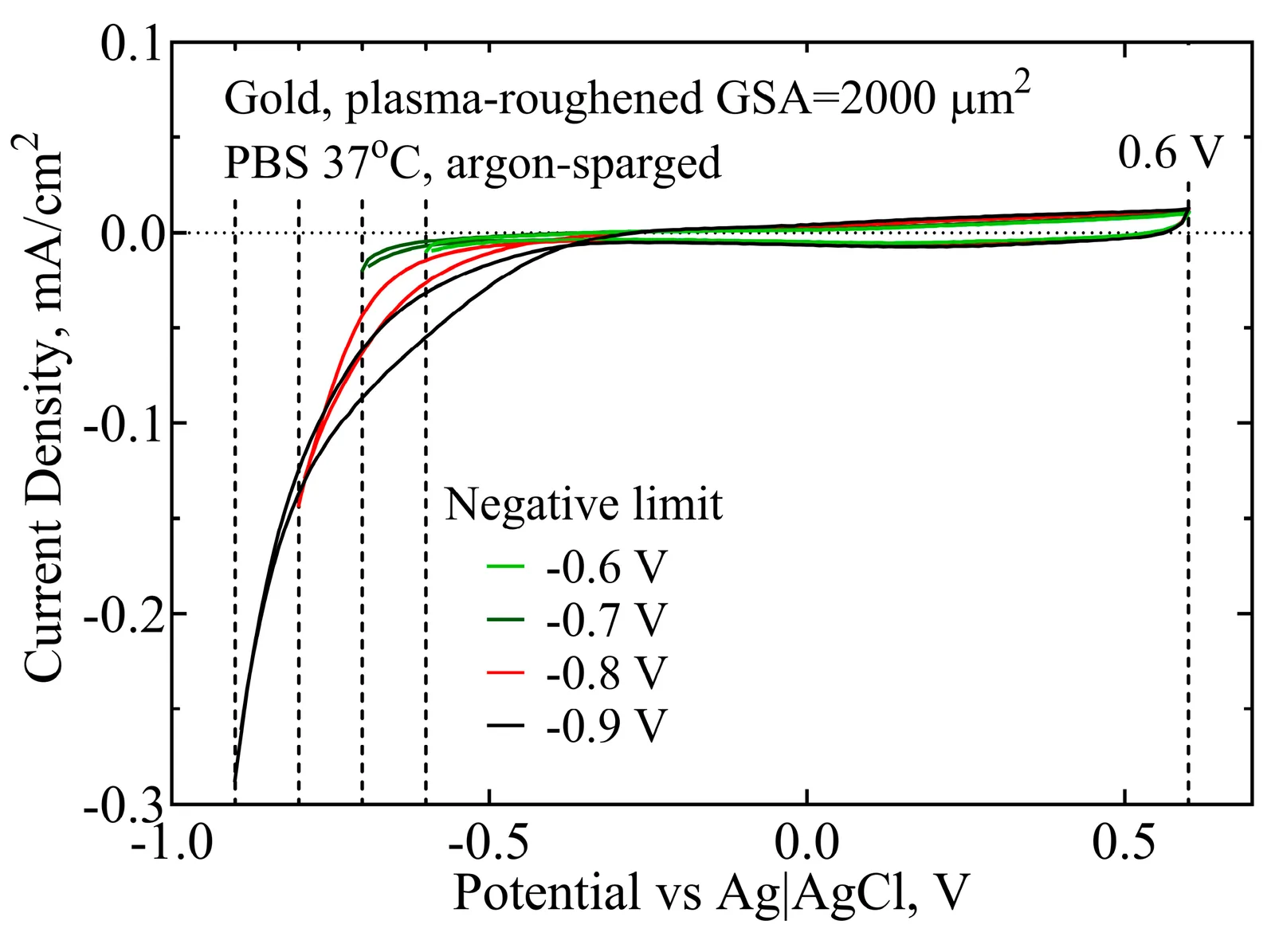
CVs of an uncoated gold electrode over negatively expanded potential ranges at a sweep rate of 50 mV/s, where the vertical dashed lines mark the negative potential limit of each CV and the common positive limit of 0.6 V, and the horizontal dotted line marks zero current density. Substantial reduction peaks emerge negative of −0.6 V, with a pronounced rise in current density between −0.7 V and −0.9 V, indicating an increasing rate of water reduction.

The observation of PEDOT:PSS degradation when pulsed to E_mc_ values <−0.6 V correlates with the asymmetry in V_acc_ and the inversion in the anodal phase of the VT (Figure 3a) that occurs at the respective E_mc_ values. Pulsed degradation, therefore, occurs in the presence of decreased PEDOT:PSS conductivity. A decrease in electronic conductivity of the PEDOT:PSS likely shifts redox reactions towards the gold/PEDOT:PSS interface, raising the possibility of reduction reactions, presumably water reduction, on the underlying gold. To investigate this possibility, CVs of uncoated gold electrodes were recorded in deoxygenated PBS across different potential ranges within the PEDOT:PSS water window (−0.9 V to 0.6 V). As shown in Figure 7, water reduction on gold begins at −0.6 V vs. Ag|AgCl and increases to a rate of 0.3 mA/cm^2^ at −0.9 V. This observation suggests that water reduction, with consequent gas formation and an increase in local pH, could be a contributing factor to PEDOT:PSS degradation.

## 4. Discussion

In this study, microelectrode arrays were coated with PEDOT:PSS by electropolymerization onto gold electrode sites with a GSA of 2000 µm^2^, a size typical of that used for neural stimulation of the cerebral cortex with indwelling microelectrodes. The as-deposited PEDOT:PSS coatings exhibited low impedance, high charge-storage capacity, and high charge-injection capacity, similar to that, at approximately the same negative potential excursion, reported by others [11,13]. Here, we investigated the stability of PEDOT:PSS with the aim of establishing charge-injection limits for long-term pulsing during electrical stimulation. Consistent with previous studies, we found the Q_inj_ of PEDOT:PSS to be higher than that of most other neural stimulation electrode coatings when using a maximum negative potential limit of −0.9 V vs. Ag|AgCl as a criterion for establishing the PEDOT:PSS charge-injection capacity. For PEDOT:PSS electropolymerized for 50 CV cycles (with a CSC_c_ of 87 ± 4 mC/cm^2^), we observed an initial charge-injection capacity of 12 ± 1 mC/cm^2^ with 200 µs/phase pulse widths. To assess the stability of PEDOT:PSS, during cathodal-first biphasic pulsing, electrodes were pulsed to E_mc_ values of −0.5 V to −0.9 V for 200 million pulses or until failure was encountered. Failure was typically abrupt and characterized by a large increase in driving voltage that saturated at the compliance limit of the stimulator (Figure 6a). Degradation of the PEDOT:PSS was confirmed by SEM, which showed complete loss of the PEDOT:PSS coating (Figure 6b). Pulsing to E_mc_ levels of −0.5 V and −0.6 V resulted in no significant degradation after 200 million pulses. However, when E_mc_ was pushed to values ≤−0.7 V, the PEDOT:PSS exhibited markedly reduced stability, with failure observed at a decreasing number of pulses as E_mc_ became more negative (Table 1).

We observed a correlation between PEDOT:PSS degradation and de-doping during pulsing. De-doping was evidenced by the V_acc_ asymmetry and anodal phase inversion at the E_mc_ values for which degradation occurred (E_mc_ <−0.6 V). An E_mc_ < −0.6 V coincides with the increased impedance of the PEDOT:PSS observed beginning at −0.5 V in biased EIS measurements. We ascribed the increase to a decreased electronic conductivity of the reduced PEDOT:PSS, which is reflected in the higher trailing access voltage at the end of the cathodal phase. A similar increase in trailing V_acc_ during cathodal pulsing can occur in activated iridium oxide (AIROF) due to a decrease in electronic conductivity as the AIROF is reduced [4]. On the anodal phase, the lower conductivity of the PEDOT:PSS results in a high leading edge V_acc_. However, as the PEDOT:PSS oxidizes and becomes more electronically conductive, V_acc_ decreases, at least over the initial portion of the anodal phase. However, while V_acc_ decreases, the polarization necessary to maintain the anodal current density at the PEDOT:PSS/PBS interface increases over the course of the anodal phase. These competing effects result in the inversion in driving voltage observed in the anodal phase at the charge densities necessary to achieve the more negative E_mc_ values. This inversion phenomenon demonstrates that redox reactions participate significantly in PEDOT:PSS charge injection using 200 µs pulse widths and current densities of 32–60 A/cm^2^ (corresponding to 6.3–12 mC/cm^2^). Notably, degradation of the PEDOT:PSS was observed during pulsing to E_mc_ values that correlated with the appearance of the inversion of the anodal VT, implicating redox reactions at potentials more negative than −0.6 V in the pulsed degradation of PEDOT:PSS.

An E_mc_ limit of −0.6 V is approximately 0.3 V positive of the reported water reduction potential of −0.9 V on PEDOT:PSS, suggesting that this reaction is not contributing to the degradation observed during pulsing to an E_mc_ of −0.7–0.8 V. Other mechanisms of degradation are possible at E_mc_ levels positive of water reduction. Volumetric changes in PEDOT:PSS that accompany reduction are a possible contributor to pulsed degradation and also consistent with the E_mc_ potential range over which degradation was observed. During reduction, cationic counterions and associated solvating water molecules enter the PEDOT:PSS to compensate for hole conduction out of the polymer. Indeed, an increase in the thickness of PEDOT:PSS coatings of 20–25% during reduction in 0.1 M NaCl, mostly occurring over a potential range of 0 V to −0.9 V vs. Ag|AgCl, has been reported [42]. Associated volumetric changes at the gold–PEDOT:PSS interface are a plausible cause of delamination observed in Figure 6b, particularly since the PEDOT:PSS is reduced and redox reactions are more likely to occur near the gold–PEDOT:PSS interface. Likewise, reduction of water on the underlying gold substrate is also consistent with the potential range over which PEDOT:PSS degradation is observed, and hydrogen gas evolution and increased pH at the PEDOT:PSS–gold interface may also drive loss of adhesion. We expect both mechanisms were active for the electrode shown in Figure 6b since water reduction would be inevitable if the PEDOT:PSS partially delaminates exposing gold polarized to ≤ −0.6 V vs. Ag|AgCl.

Regardless of the specific mechanism of PEDOT:PSS degradation, interfacial failure at the PEDOT:PSS and gold interface is evident when the coatings are pulsed to potentials more negative than −0.6 V vs. Ag|AgCl. This observation suggests that using the water reduction potential on PEDOT:PSS overestimates the charge-injection capacity for long-term pulsing. A more conservative limit of −0.6 V vs. Ag|AgCl, which avoids reduction-driven de-doping of the PEDOT:PSS, would seem a better limit for estimating maximum charge-injection capacity. For the PEDOT:PSS films investigated in the present study, this results in a Q_inj_ maximum of 3.6 ± 0.2 mC/cm^2^ for films with a CSC_c_ of 73–87 mC/cm^2^, notably lower than earlier reports of 15 mC/cm^2^ that used a −0.9 V vs. Ag|AgCl as the negative limit [4]. Similar to that reported by others [29,35], we found surface roughening of the gold electrode sites prior to PEDOT:PSS deposition to be effective in improving adhesion, although degradation was not avoided at negative potentials (<−0.6 V Ag|AgCl) during current pulsing. A controlled comparison between roughened and unroughened substrates was not performed due to frequent delamination of PEDOT:PSS from unroughened gold during initial testing. Besides physical surface roughening, many approaches to improving adhesion and pulsing stability involving counterions other than PSS^-^ [31] and the incorporation of a second electroactive phase, such as carbon nanotubes [15], have been investigated, with notable improvements in stability. A comprehensive tabulation of PEDOT-based electrode coatings has been provided by Zheng et al. [39] in their 2021 review. The CICs of the PEDOT-based coatings ranged from 1.36 to 11.6 mC/cm^2^, primarily for a 200 µs pulsewidth. Using a −0.6 V vs. Ag|AgCl, Green et al. [43], using laser-roughened platinum substrates, established charge limits for PEDOT doped with paratoluene sulfonate or ClO_4_^-^ that increased from 1.3 mC/cm^2^ to 3.7 mC/cm^2^ as the pulsewidth was increased from 100 µs to 800 µs Their PEDPT:PSS coatings ranged from 0.8 to 1.8 mC/cm^2^ over the same pulsewidth range. As an alternative to plasma or laser roughening, Boehler et al. [44] employed sputtered films of iridium oxide (SIROF) or nanostructured Pt as a substrate for PEDOT electrodeposition and reported notable improvements in PEDOT:PSS adhesion compared with unroughened Pt, using CV cycling to a negative potential limit of −0.6 V vs. Ag|AgCl. In vivo stability of passive SIROF/PEDOT:PSS probes for neural recording was demonstrated over a 5-month implantation period [34].

Observations from the present study, combined with extensive prior reports on the stability of PEDOT-based coatings, implicate substrate adhesion and volumetric changes during reduction as factors compromising stability for neural recording and stimulation. Any deleterious effect of substrate electrochemical reactions, such as water reduction, remain to be explored in detail but will be of concern for stimulation that polarizes the PEDOT coatings to potentials negative of the water reduction potential of the substrate. For stimulation electrodes, at least, it would be of interest to explore PEDOT coatings stabilized with a rigid, non-compressible second phase, such as CNTs, pulsed to potentials negative of −0.6 V vs. Ag|AgCl. Structural stability provided by the rigid phase may reduce susceptibility to degradation associated with de-doping and volume changes. In any case, observations of asymmetry in the access voltage (ΔV_acc_) of the cathodal phase and an anodal phase inversion are correlated with pulsed degradation of PEDOT-PSS and should raise concerns about stability during stimulation. It remains to be determined whether the addition of CNTs, alternative dopants to PSS^-^, or the various other approaches can stabilize PEDOT-based coatings polarized more negative than −0.6 V vs. Ag|AgCl.

A clear limitation of the present study is the number of pulses, 200 million, used to assess stability. In clinical neural stimulation applications, pulse repetition rates of at least 50 Hz are common, representing only ~46 days of continuous stimulation to reach 200 million pulses. More extended periods of pulsing at −0.6 V are necessary to establish Q_inj_ for long-term applications. Lastly, the in vivo stability of PEDOT-based coatings during pulsed stimulation needs to be assessed in long-term chronic studies.

## 5. Conclusions

This study suggests that PEDOT:PSS coatings on plasma-roughened gold provide stable charge injection for neural stimulation when operated within defined potential limits. Films maintained stability at an E_mc_ of –0.6 V and Q_inj_ = 3.6 mC/cm^2^ for 200 million current pulses without degradation. In contrast, pulsing to more negative potentials (<–0.6 V) significantly compromised stability, leading to elevated impedance, reduced charge-storage capacity, and structural deterioration. These observations suggest –0.6 V vs. Ag|AgCl as a threshold for the 200-million pulse operation of PEDOT:PSS coatings deposited on roughened gold. Features of the voltage transient response, access voltage asymmetry and anodal phase inversion were also coincident with degradation. Overall, this work identifies key electrochemical limits for PEDOT:PSS stability and may provide insight into the development of more stable conducting polymer coatings in bioelectronic applications.

## Figures and Tables

**Table 1:** CSC_c_ and impedance at 1 kHz of PEDOT:PSS electrodes before and after stimulation at different E_mc_ levels. The maximum number of pulses for the long-term stimulation experiments was capped at 200 million pulses. (Data are mean ± sd, *n* = 4).

E_mc_,V	CSC_c_ Pre-Stim mC/cm^2^	|Z| 1 kHz Pre-Stim, kΩ	CSC_c_ Post-Stim mC/cm^2^	|Z| 1 kHz Post-Stim, kΩ	Pulses, Millions	Q_inj_ mC/cm^2^
−0.5	79 ± 19	4.5 ± 0.1	70 ± 16	3.54 ± 0.14	no failure	3.1 ± 0.2
−0.6	73 ± 11	5.2 ± 0.2	67 ± 12	4.6 ± 0.4	no failure	3.6 ± 0.2
−0.7	90 ± 15	4.9 ± 0.2	12 ± 19	332 ±191	174 ± 36	6.3 ± 0.8
−0.8	97± 36	4.7 ± 0.9	0.54 ± 0.7	496 ± 211	48 ± 39	8.3 ± 1.9
−0.9	87 ± 4	4.7 ± 0.3	0.05 ± 0.05	684 ± 128	2.5 ± 2.5	12 ± 1

## Data Availability

The raw data supporting the conclusions of this article will be made available by the authors on request.
